# A modified evidence-based practice- knowledge, attitudes, behaviour and decisions/outcomes questionnaire is valid across multiple professions involved in pain management

**DOI:** 10.1186/s12909-014-0263-4

**Published:** 2014-12-14

**Authors:** Qiyun Shi, Bert M Chesworth, Mary Law, R Brian Haynes, Joy C MacDermid

**Affiliations:** Health & Rehabilitation Sciences, Western University, Room 1014, Elborn College, 1201 Western Road, London, ON N6G 1H1 Canada; Hand and Upper Limb Centre Clinical Research Laboratory, St. Joseph’s Health Centre, 268 Grosvenor St, London, ON N6A 3A8 Canada; School of Physical Therapy, Western University, London, ON N6G 1H1 Canada; Department of Epidemiology and Biostatistics, Western University, London, ON N6G 1H1 Canada; The School of Rehabilitation Sciences, McMaster University, Hamilton, ON L8S 4L8 Canada; Department of Clinical Epidemiology and Biostatistics and Department of Medicine, McMaster University, Hamilton, ON L8S 4K1 Canada

**Keywords:** Evidence-based, Scale, Self-reported, Validation, Clinician

## Abstract

**Background:**

A validated and reliable instrument was developed to knowledge, attitudes and behaviours with respect to evidence-based practice (EBB-KABQ) in medical trainees but requires further adaptation and validation to be applied across different health professionals.

**Methods:**

A modified 33-item evidence-based practice scale (EBP-KABQ) was developed to evaluate EBP perceptions and behaviors in clinicians. An international sample of 673 clinicians interested in treatment of pain (mean age = 45 years, 48% occupational therapists/physical therapists, 25% had more than 5 years of clinical training) completed an online English version of the questionnaire and demographics. Scaling properties (internal consistency, floor/ceiling effects) and construct validity (association with EBP activities, comparator constructs) were examined. A confirmatory factor analysis was used to assess the 4-domain structure EBP knowledge, attitudes, behavior, outcomes/decisions).

**Results:**

The EBP-KABQ scale demonstrated high internal consistency (Cronbach’s alpha = 0.85), no evident floor/ceiling effects, and support for a priori construct validation hypotheses. A 4-factor structure provided the best fit statistics (CFI =0.89, TLI =0.86, and RMSEA = 0.06).

**Conclusions:**

The EBP-KABQ scale demonstrates promising psychometric properties in this sample. Areas for improvement are described.

**Electronic supplementary material:**

The online version of this article (doi:10.1186/s12909-014-0263-4) contains supplementary material, which is available to authorized users.

## Background

Evidence-based practice (EBP) is defined as the integration of the best research evidence with patients’ interests and clinical circumstances in decision making [1]. As EBP is associated with improved clinical decision-making and patient care [2], health professional organizations have advocated for increased training in EBP for all health care professionals at all levels of education [3,4]. Understanding how EBP is understood and implemented across different health professionals can identify educational needs and outcomes, and predict where new research evidence is more likely to be implemented. As such, a validated and reliable instrument is required to evaluate an individual’s perceptions of EBP.

A systematic review [5] which studied 104 instruments on EBP suggested that evaluation of EBP could be divided into the following definable components: EBP knowledge, attitudes toward EBP, application/use of EBP and practitioners’ EBP behaviors in the clinical setting. *Knowledge about EBP* means that clinicians have knowledge of fundamental EBP concepts and terminology and concepts related to quality or levels of evidence. It also includes the ability to search the literature and critically appraise the evidence for its validity, impact and applicability. *Attitude toward EBP* includes the intuitive appeal of EBP, the likelihood of adopting EBP given professional requirements to do so, openness to new practices, and the perceived divergence between research-based/academically developed interventions versus current practice [6]. *Application and use of EBP* refers to whether health professionals are able to apply their EBP knowledge to the specific clinical scenarios. This includes: capability to generate clinical question(s) regarding disease prevention, diagnosis and management as well as implementation of evidence with integrity of clinical circumstances. EBP b*ehaviors* refer to practitioners’ performance of the instrumental activities associated with EBP such as searching and obtaining higher quality evidence in their own practice.

Although the rise of EBP awareness has led to the development of instruments to assess its integration into clinical practice, there are gaps in the evidence supporting these tools [5]. There is a lack of empirical data that can be applied to a wider range of experience and types of clinicians, in particular nurses and allied health professionals [3]. Moreover, as most scales have targeted samples with minimal experience in clinical practice, the questionnaires may not accurately reflect the perception of EBP by clinicians who have been practicing in different clinical settings.

Among available scales, one that has taken a multi-dimensional approach and shown early promise is the *The knowledge, attitude and behavior questionnaire* (KAB) originally developed by Johnson and colleagues [7]. The KAB scale was designed to evaluate EBP teaching and learning in the undergraduate medical education setting. With permission from the developers, two study authors (JMD and ML) developed a modified KAB scale (EBP-KABQ), to be applicable to health professionals other than physicians using expert review and pilot testing. This process resulted in removal of items that were perceived by users as redundant or unclear.

The goal of this study was to validate the modified scale (EBP-KABQ) for use in a multidisciplinary group of clinicians by determining: (1) Scaling properties- internal consistency, floor/ceiling effects, and (2) Construct validity- based on predetermined hypotheses on the relationship of subcomponents of EBP, and (3) Structural validity: the integrity of a 4-domain structure based on confirmatory factor analysis.

## Methods

The EBP-KABQ incorporates 33 items in four domains of EBP: knowledge (8 items, 6 ordinal items), attitudes (14 items, 14 ordinal items), behaviour (8 items, 5 ordinal items) and outcomes/decisions (3 items, 3 ordinal items) (KABQ). The knowledge items retain a 7-point Likert scale with lower scores indicating a lower level of EBP knowledge. The Attitudes towards EBP items retain a 7-point Likert scale. High scores indicate positive attitude after several items were reversely scored. For EBP behaviour, lower scores indicate a lower frequency of using EBP in current practice. A 6-point Likert scale is used for responses to the items in the outcomes/decisions domain. Lower scores indicate unfavorable patient outcomes and poor clinical evidence-based decision making. Detail of the EBP-KABQ scale and a summary of the changes to original scale are presented in Additional files 1 and 2.

### Subject recruitment and data collection

All participants were recruited from a clinical trial assessing use of pain research evidence about pain [8]. Eligible practitioners were (1) physicians, nurses, occupational therapists (OTs), physical therapists (PTs), or psychologists who were currently working in clinical practice at least one day/week; (2) fluent in English; (3) able to access a computer at home or at work that provided unrestricted access to the World Wide Web; (4) possessed an active email account;(5) consent to participate in this research studyA total of 870 clinicians met the inclusion criteria and were invited to participate. From August 2011 to February 2013, 673 clinicians (physicians, nurses, OTs/PTs, psychologists etc.) completed an online EBP-KABQ scale prior to receiving new pain information. Demographic and practice characteristics were also obtained. The study received Ethics Approval from the McMaster University Research Ethics Board.

### Data analysis

Quality checks, descriptive statistics and checks for normality were completed prior to analysis. Item 33 “I don’t use evidence-based practice for another reason (specify)” was removed from the analyses because the specified reason varied across respondents, making it a nonstandard item. Therefore, 27 ordinal items across the following four domains of EBP were analyzed in this study: knowledge (n = 6 items), attitudes (n = 13 items), behavior (n = 5 items) and outcomes/decisions (n = 3 items).

### Scaling properties (internal consistency and floor/ceiling effects)

Internal consistency reliability scores were assessed for both the full EBP-KABQ scale and its corresponding 4 subscales using Cronbach’s alpha, where >0.7 was considered as minimum [9] and >0.9 was desirable [10]. Scaling properties such as floor/ceiling effects, which was observed in >15% of scores at minimum or maximum scale/subscale were also assessed [11].

### Construct validation

Four hypotheses were tested to assess the construct validation of EBP-KABQ scale. First, we hypothesized that the mean item score in “knowledge” would be higher than those in “behaviour”, “outcomes/decisions” and “attitude” domains because knowledge is considered a necessary precursor, but not a sufficient guarantee, for changes in practice and outcomes. Secondly, we hypothesized that the domain of “outcomes/decisions” would be more strongly correlated to the other 3 domains since it focuses on how EBP influences the decision making process. Thirdly, we hypothesized that EBP-KABQ subscale scores would be correlated with corresponding EBP activities assessed by relevant open ended questions. For example, the frequency that clinicians search for evidence should be correlated with subtotal score of “behaviour” to a greater extent than other domains such as “knowledge” or “EBP outcomes/decisions”. Finally, we hypothesized that following demographic variables would be associated with total EBP-KABQ scale score in the multivariate modeling: age, highest level of education, and possession of advanced clinical training since these have been suggested in the literature on EBP. Details of all construct validity testing and a priori hypotheses are provided in the Results section.

### Structural validity

Confirmatory factor analysis (CFA, maximum likelihood estimation) was conducted to examine our proposed 4-domain model. Four conceptual domains of EBP (knowledge, attitudes, behavior and outcomes/decisions) were tested as second-order factors (latent variables) based on the originally defined conceptual framework. We evaluated the model fit with a number of goodness-of-fit statistics including Root Mean Square Error of Approximation (RMSEA) <0.06 (ideal) and <0.08 (acceptable), comparative fit index (CFI) ≥0.90–0.95 (acceptable), Tucker Lewis Index (TLI) ≥0.90–0.95 (acceptable) and Chi-square test (P > 0.05, acceptable) [12-15]. We considered RMSEA, CFI and TLI as primary statistics because Chi-square is vulnerable to a large sample size (sample size > 300) [12]. We also examined modification indices to identify the potential to improve the model fit. We modified our model when it was indicated by theoretical and statistical findings [16]. We considered standardized coefficients (i.e., factor loadings) ≥0.30 (p < 0.05) as ‘representing’ a hypothesized dimension [17].

All analyses except CFA were conducted by SAS (version 9.3, SAS Institute Inc, Cary, NC, USA). We used IBM SPSS v20 Amos statistical software for CFA.

## Results

### Sample characteristics

In total, 673 health professionals completed EBP-KABQ questionnaire. The description of demographic characteristics is presented in Table 1. Half of participants were age 45 or younger. Nearly half of clinicians were OTs or PTs, while 1/4 were nurses and 1/5 were physicians. One quarter of the sample had more than 5 years of clinical training; and they had a mean time in clinical practice of almost 18 years. Most participants practiced in an urban setting, while 15% were in a rural practice area.

**Table 1:** **Characteristics of 673 participants of EBP-KABQ study**

**Characteristics**	**N (%)**
**Age**	
20–35	178 (26.4)
36–45	158 (23.4)
46–55	221 (32.8)
56+	116 (17.2)
**Clinical designation**	
MD	131 (19.5)
OT/PT	326 (48.4)
RN	127 (18.8)
RPsych or CPsych	52 (7.7)
Others	37 (5.5)
**Highest education level**	
Diploma/BA	234 (34.8)
MA/MSC	222 (33.0)
MD	122 (18.1)
Ph.D.	95 (14.1)
**Received advanced clinical certifications**	364 (54.1)
**Years of clinical training**	
Less than 2 years	190 (28.2)
2–5 years	295 (43.8)
Above 5 years	188 (27.9)
**Location of practice**	
Urban	463 (68.8)
Rural	101 (15.0)
Both	109 (16.2)

Years of clinical experience: Mean = 17.96 years (SD = 11.23 years; range = 0–52).

### Scaling properties (internal consistency and floor/ceiling effects)

Overall, EBP-KABQ scale achieved acceptable satisfactory internal consistency (Cronbach’s alpha α = 0.85) although the subscale of “knowledge” still showed marginal acceptable internal consistency with Cronbach’s alpha = 0.66 after removal of item 3. However, this was improved compared to the original 6-item “knowledge” subscale (Cronbach’s alpha = 0.56). This finding supported the decision to remove item 3 (“Clinical trials and observational methods are equally valid in establishing treatment effectiveness”).

Table 2 presents a summary of the item-level properties of EBP-KABQ. The mean and median total score of EBP-KABQ scale was 117.93 (SD: 15.10) and 118 respectively, with no floor/ceiling effects detected. The mean scores of four subscales ranged from 11.22 to 64.58. Similarly, no obvious floor/ceiling effects were observed in all four subscales although some individual items particularly in “knowledge” presented a ceiling effect.

**Table 2:** **Descriptive statistics of the EBP-KABQ scale, scaling properties and internal consistency (n = 673)**

**Scale**	**Item**	**Item mean(SD)**	**Median**	**Floor%**	**Ceiling%**	**Subscale mean(SD)**	**Floor%**	**Ceiling%**	**Cronbach’s alpha at subscale/total level**
Knowledge-5 items	EBP-KABO1	5.79 (1.02)	6.00	0.1	**23.8**	29.57(3.62)	0.1	1.5	0.66
	EBP-KABO2	6.01 (0.99)	6.00	0.3	**34.0**				
	EBP-KABO4	5.44 (1.41)	6.00	1.6	**25.6**				
	EBP-KABO5	6.08 (1.06)	6.00	0.6	**41.0**				
	EBP-KABO6	6.25 (1.12)	7.00	0.6	**55.0**				
Behaviour-5 items	EBP-KABO9	3.14 (1.16)	3.00	1.2	**15.6**	11.22(4.28)	0.1	0.3	0.77
	EBP-KABO10	2.02 (1.15)	2.00	**17.5**	3.1				
	EBP-KABO11	2.42 (1.22)	2.00	7.0	5.8				
	EBP-KABO12	1.66 (1.11)	2.00	**25.6**	1.6				
	EBP-KABO13	1.98 (1.31)	2.00	**34.3**	5.9				
Outcome/Decision-3 items	EBP-KABO17	4.56 (0.94)	5.00	1.0	12.0	12.56(2.52)	0.3	0.4	0.83
	EBP-KABO18	4.11 (1.04)	4.00	1.9	4.8				
	EBP-KABO19	3.88 (0.93)	4.00	0.3	3.4				
Attitude-13 items	EBP-KABO20	4.17 (0.69)	4.00	1.3	**32.1**	64.58(8.99)	0.1	0.3	0.75
	EBP-KABO21	5.20 (1.53)	5.00	**0.9**	**25.7**				
	EBP-KABO22	3.94 (1.59)	4.00	3.9	4.2				
	EBP-KABO23	4.18 (1.56)	4.00	3.6	8.5				
	EBP-KABO24	4.88 (1.59)	5.00	1.8	**18.6**				
	EBP-KABO25	4.81 (1.39)	5.00	0.9	11.9				
	EBP-KABO26	6.22 (1.02)	7.00	0.7	**50.1**				
	EBP-KABO27	4.72 (1.51)	5.00	2.5	9.4				
	EBP-KABO28	5.77 (0.99)	6.00	0.3	**25.3**				
	EBP-KABO29	5.99 (0.98)	6.00	0.3	**36.4**				
	EBP-KABO30	3.80 (1.51)	4.00	10.1	2.1				
	EBP-KABO31	5.26 (1.66)	6.00	1.9	**31.4**				
	EBP-KABO32	5.66 (1.41)	6.00	0.6	**38.0**				
MEBP-26 items	Full version	-----	118.00	-----	-----	117.93(15.10)	0.1	0.1	0.85

Bold indicated floor or ceiling effect. Item 3 was removed from the scale based on factor structure.

### Construct validity

Details of the construct validity testing and a priori hypotheses were provided in Table 3. As we expected, mean item score in “knowledge” was 5.91, significantly higher than the rest of the domains (p < 0.05). Our constructed hypotheses were supported in that the correlation coefficients between “outcomes/decision” and “knowledge”, “behaviour” and “attitude” were 0.54, 0.40 and 0.57 respectively, which were higher correlations than observed between other subscales. Construct validity was also supported in that there was a significant relationship between the frequency of searching reported by clinicians and the “behaviour” score, with correlation coefficient ranges from 0.32 to 0.41 (hypothesis 3). Regression analyses supported our a priori hypothesis that health professionals who had higher levels of education (β = 4.63, P < 0.01), longer years in clinical training (β = 2.36, P < 0.01) and possession of advanced clinical training (β = 4.37, P < 0.01) were more likely to use EBP (Table 4). Although younger age was related to EBP practice in the direction anticipated, it did not reach statistical significance (β = −0.32, P = 0.06).

**Table 3:** **Results of construct validity against a series of theoretical constructs**

**Theoretical constructs**	**A priori hypotheses**	**Results**
1. EBP knowledge is more easily affected than other other aspects of EBP	Mean item score in “knowledge” > other domains	**Knowledge**: 5.91
		Behaviour: 2.24
		Outcome/Decision : 4.18
		Attitude: 4.96
2. “Outcome/Decision” is correlated to other 3 domains	Correlation coefficients between “outcome” and “knowledge”/“application”/“attitude” > other correlation coefficients.	r_outcome-knowledge=0.54_*, r_outcome-behaviour=0.40_*, r_outcome-attitude=0.57_*;
		r_attitude-knowledge=0.41_*, r_knowledge-application=0.33_*, r_application-attitude=0.26_*;
3. MEBP subscale scores are correlated with corresponding EBP activities	Correlation coefficients between “application” and 3 external questions evaluating EBP application > other correlation coefficients.	r_application-Q1=0.32_*, r_knowledge-Q1=0.19_*, r_outcome-Q1=0.28_*; r_attitude-Q1=0.19_*;
		r_application-Q2=0.41_*, r_knowledge-Q2=0.24_*, r_outcome-Q2=0.30_*; r_attitude-Q2=0.19_*;
		r_application-Q3=0.35_*, r_knowledge-Q3=0.24_*, r_outcome-Q3=0.26_*; r_attitude-Q3=0.16_*;
4. Demographic variables would be associated with total MEBP scale score	Age, highest education level, possession of advanced clinical training are significant factors are associated with in multivariate modeling	Adjusted β coefficients of following variable:
		Age: β = −0.32
		Higher education level (ref: diploma/BA): β =4.63*
		Years of clinical training (ref: less than 2 years): β =2.36*
		Advanced clinical training (ref: No): β =4.37*
		Practice setting (ref: urban): β =1.87*

*P < 0.05.

Q 1: How often do you now look up evidence immediately before, or during patient treatment visit per week?

Q 2: How many hours do you spend looking up evidence per week?

Q 3: How many hours do you spend reading new research evidence per week?

**Table 4:** **Unadjusted and adjusted linear regression coefficients for EBP-KABQ total score**

	**Unadjusted**			**Adjusted**		
**Characteristics**	**B**	**SD**	**P value**	**B**	**SD**	**P value**
Age (years)	−0.32	0.59	0.60	--	--	--
Education (ref: diploma/BA)	5.08	0.73	<0.01*	4.63	0.73	<0.01*
Clinical designation (ref: MD)	0.06	0.06	0.27	--	--	--
Years of clinical training (ref: less than 2 years)	3.57	0.73	<0.01*	2.36	0.81	<0.01*
Advanced clinical training (ref: No)	5.68	1.23	<0.01*	4.37	1.21	<0.01*
Practice setting (ref: urban)	1.24	0.82	0.13	1.87	0.79	0.02*
Constant	--	--	--	106.2	2.43	<0.01*

*P < 0.05, variables were selected if p value <0.2 in univariate model.

### Structural validity

**Figure 1:**
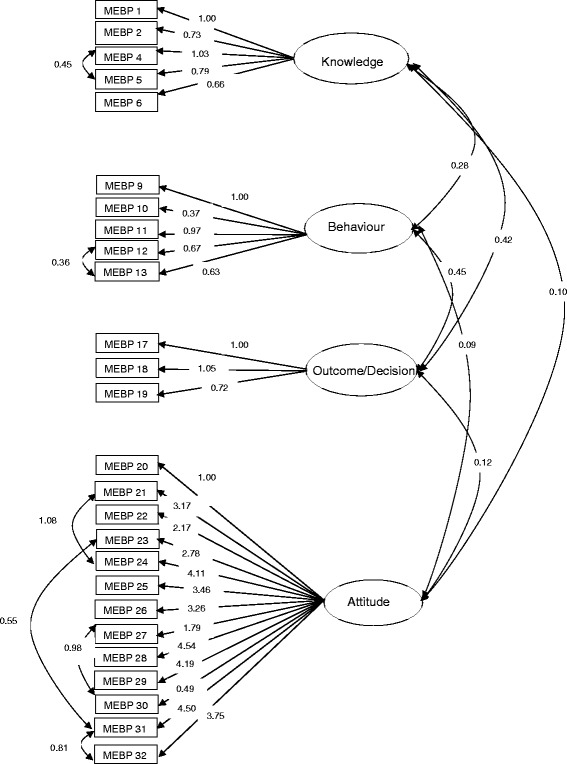
**Standardized parameter estimates for the refined EBP-KABQ factor structure model.** Rectangles represent the scale items and ellipses represent the proposed factor constructs. Values on the single-headed arrows leading from the factors are standardized factor loadings. Values on the curved double-headed arrows between rectangles are correlations between error terms. Values on the curved double-headed arrows between ellipses are correlations between latent variables.

The Initial second-order model demonstrated poor model fit (x^2^ = 1838.24, df = 269, P < 0.001, CFI = 0.73, TLI = 0.70, RMSEA = 0.093). Modification indices suggested overall model fit would be improved by adding the correlation of six pairs of error terms (item 4 & 5 within “knowledge”, 12 & 13 in “application”, 21 & 24, 23 & 31, 27 & 30, and 31 & 32 in “attitude”). After the modification was executed, statistical fit of the model was improved to as follows: ×^2^ = 1205.20, df = 312, P < 0.001, CFI = 0.86, TLI = 0.84, RMSEA = 0.065. Although the overall fit improved, model fit indices especially CFI and TLI were still inadequate. We observed factor loading (β = 0.05) of the item 3 (“Clinical trials and observational methods are equally valid in establishing treatment effectiveness”) was significantly lower than the other five items on the dimension of knowledge. After removing this item from the scale, goodness-of-fit statistics improved to ×^2^ = 1056.65, df = 287, P < 0.001, CFI = 0.89, TLI = 0.86, RMSEA = 0.06 (Figure 1) which was very close to our a priori threshold (CFI/TLI ≥ 0.90, RMSEA < 0.08).

## Discussion

This study provided support for the use of a modified EBQ-KABQ questionnaire to understand different aspects of EBP knowledge, attitudes, behavior and outcomes/decisions in a variety of healthcare professionals with respect to EBP. We confirmed that the 26 ordinal items in the modified EBP-KABQ exhibit a four-domain construct consistent with the proposed four aspects of EBP. Our scale was modified based on our need to change wording to make the scale more broadly applicable to different disciplines since the original version targeted medical students. We also made changes based our experiences in pilot testing the measure since an expert committee and pilot users found some items to be redundant or difficult to understand. Our work builds on that of the developers who targeted medical trainees by providing a more broadly applicable and validated version. The newly proposed subscale construct of “outcomes/decisions” contains the items previously termed “future use” in the original scale. Outcomes/decisions more accurately reflect the item content and the targeting of the EBP-KABQ. Whereas, as the original instrument was focused on trainees who might be responding about future use, experienced clinicians will be reporting how they use EBP in current clinical decision-making and whether they attribute better outcomes to their evidence-based decisions. This domain is considered an important aspect of self-reported EBP since its focuses on the impact on practice and outcomes. We found the “outcomes/decisions” domain was moderately correlated with the other three domains, suggesting it played a role in perception of EBP. The shorter measure has improved measurement characteristics, retains conceptual domains and may be save administration time.

We found the EBP-KABQ scale demonstrates promising psychometric properties when measuring EBP in practicing health professionals because our analysis supported hypotheses posed for construct validity, and we found appropriate scaling properties. The overall Cronbach’s alpha (0.85) was superior to that of the original KAB scale (0.75) which may be attributed to deletion of problematic items.

The correlation between the knowledge and attitude/application domains was relatively weak. This suggests that these are relatively distinct domains. One explanation for this low correlation may be that increased focus on EBP in entry-level and post-professional education may have had more impact on knowledge than on attitudes and application of EBP [18]. However, measurement error may also have contributed. We observed lower internal consistency of the “knowledge” domain compared to other subscales and compared to the original KAB [7]. Low internal consistency suggested that the six items within the construct of “knowledge” were not adequately correlated. As item 3 (Clinical trials and observational methods are equally valid in establishing treatment effectiveness) demonstrated low factor loading to domain of “knowledge”, we questioned the content validity of this item. One explanation for this misfit item could be that clinicians might have confused the words “observational study” with “clinical observation”. However, we suspect that controversy over the “level of evidence” or “quality” of observational studies [19,20] may have contributed to misfit on this item. In fact, more recent trends in evidence rating have acknowledged large observational studies as offering high quality evidence [21]. Respondents may value large observational studies more than small trials and not endorse this item despite strong knowledge of EBP. Since this item does not appear to reflect the domain of “knowledge”, and did not fit in CFA, we proposed removal. We suggest caution when using the “knowledge” subscale on its own to evaluate EBP knowledge, as further investigation is warranted to improve this sub-scale.

We found items in EBP knowledge skewed to the high extreme, whereas the others subscales did not demonstrate this. As evidence-based practice has become accepted around the world, it is now commonly integrated in the clinical training of many professionals [22]. Hence, knowledge about what evidence-based practice is, becomes prevalent over time [9]. Our finding may be explained by the fact that traditional evidence-based training focuses on providing knowledge to help practitioners enhance their techniques and skill level when searching and appraising evidence [23-27] but less consistently focuses on implementation behaviours for integrating EBP into daily clinical activities nor resolving attitudinal barriers towards EBP [28-30]. For instance, clinicians may enhance their knowledge of methods to find and appraise evidence, including the importance of systematic reviews in the evidence-based practice paradigm, but not be willing to able to incorporate this into their day-to-day clinical decision-making. Continuing medical education events often focus on providing content knowledge rather than active approaches, although the latter is more effective in promoting behavior change [31]. This may contribute to the findings observed in the study.

We found several factors were associated with better uptake of EBP. People with a higher level of education, more years of training, completion of advanced clinical training and those practicing in rural areas reported a greater willingness to implement EBP in their daily practice. Our findings were consistent with other studies [32-34] that also found health professionals with a higher level of education were more willing to adopt evidence-based practice. On the other hand, our finding that age was not a factor influencing EBP is in contrast to the literature [32,34] that shows recent graduates are more likely to accept EBP than clinicians who are older. Our findings were narrowly insignificant (p < 0.06) suggesting a small effect of age may not have reached significance. However, age may be less important over time as EBP spreads through post-professional training.

Out findings suggest clinicians who practices in rural areas are more amenable to EBP which was an unexpected finding. This may be explained by several reasons. First, clinicians in rural areas are more likely to seek evidence because they have fewer colleagues in their work environment to discuss clinical issues when questions emerge in day-to-day practice. As a consequence, they would be more accustomed to going to the Internet looking for online evidence as a medical resource. Secondly, geography is no longer a barrier for clinicians to acquire evidence based education. McColl [35] reported only 16% of physicians in England received official education regarding literature search techniques. Therefore, clinicians in rural areas may have access to gaining skills in EBP during their professional training, or through other avenues and be motivated to use these skills to solve their clinical questions.

Our study has some limitations. While it was a strength that we had different professions and a geographically diverse sample, we were unable to explore how contextual factors contributed to our findings. Local differences regarding the EBP training, culture and language among these participants were not captured in our data collection and we could not test for the influence of many potential covariates and limited covariate testing to factors suggested as important in the literature. However, h a broader sample improves the generalizability of our findings. Since the survey was only offered in English, our findings may not represent contexts where English was not a common language. A further consideration is that the data were self-reported. We have no external criterion to examine whether the self-reported evidence-based practice behaviors are consistent with actual practice. The impact of EBP decisions on patient outcomes may be overestimated if physicians overestimate their ability to improve outcome [36]. Studies of EBP that measure patient outcomes by patient-report or objective measures are preferable indicators of the impact of EBP, but can be challenging to measure [37,38]. We had to make decisions about deletion of items based on expert review and statistical performance. Studies of the reasons for poor item performance that included qualitative techniques such as cognitive interviewing may have identified ways to reform problematic items or captured new concepts. However, since our goal was to stay true to the original KABQ, if possible, our approach was reasonable. Finally, since our sample was derived from clinicians interested in pain, it may not reflect all. Since pain is the most common patient complaint and one relevant across different professions it represented an ideal context to test the EBP-KABQ across professions and contexts.

## Conclusion

This study provides evidence in a large sample of experienced clinicians from a range of professions interested in pain management that the EBP-KABQ can be used to assess four domains of EBP: Knowledge, attitude, behavior, outcomes/decisions.

## Additional files

Additional file 1:
**Modified Knowledge/Attitudes/Behaviours Questionnaire.**
Additional file 2:
**Comparison of EBP-KABO to KAB questionnaire developed by Johnson et al.**

## Abbreviations

CFA: Confirmatory factor analysis
CFI: Comparative fit index
EBP: Evidence–based practice
KAB: Knowledge, attitudes, behavior
RMSEA: Root mean square error of approximation
TLI: Tucker Lewis index
