# Supplementary material for: A modified evidence-based practice- knowledge, attitudes, behaviour and decisions/outcomes questionnaire is valid across multiple professions involved in pain management
Source: BMC Med Educ. 2014 Dec 14;14:263. doi: 10.1186/s12909-014-0263-4 (PMC4272818; doi:10.1186/s12909-014-0263-4)
Supplement: Additional file 1: — Modified Knowledge/Attitudes/Behaviours Questionnaire. [file 12909_2014_263_MOESM1_ESM.doc]

|  |
| --- |

Appendix 1

Modified Knowledge/Attitudes/Behaviours Questionnaire

| This is a survey designed to evaluate various aspects of Evidence based practice. Please answer truthfully (i.e. Do not tell us what you THINK we want to hear, rather tell us what YOU really believe) and complete all the questions.  All responses will be treated in strict confidence and seen only by independent research assistants. All individual identities will be masked and the analysis of the data will be blinded. Only the aggregate results will be published.  Thank you for your participation. |
| --- |

## For the purposes of the rest of this questionnaire, we define Evidence-Based Practice as follows:

Using/Applying evidence-based practice means the conscientious, explicit and judicious use of current best evidence in making decisions about the care of individual patients. That is, looking up, appraising and applying both basic, factual information as well as disease- and condition-specific evidence.

Please circle the most appropriate response:

|  | **Strongly Agree** | **Moderately Agree** | **Somewhat Agree** | **Neutral** | **Somewhat Disagree** | **Moderately Disagree** | **Strongly Disagree** |
| --- | --- | --- | --- | --- | --- | --- | --- |
| 1. I am confident in my ability to use evidence-based practice. | 7 | 6 | 5 | 4 | 3 | 2 | 1 |

|  | |  | | | | | | | | | | | | | | |
| --- | --- | --- | --- | --- | --- | --- | --- | --- | --- | --- | --- | --- | --- | --- | --- | --- |
|  | | Please indicate how much you agree/disagree with the following statements. | | | | | | | | | | | | | | |
|  |  | | | | **Strongly Agree** | | **Moderately Agree** | | **Somewhat Agree** | | **Neutral** | | **Somewhat Disagree** | | **Moderately Disagree** | **Strongly Disagree** |
| 2. | Using evidence-based practice increases the certainty that the proposed treatment is effective. | | | | 7 | | 6 | | 5 | | 4 | | 3 | | 2 | 1 |
| 3. | Clinical trials and observational methods are equally valid in establishing treatment effectiveness. | | | | 7 | | 6 | | 5 | | 4 | | 3 | | 2 | 1 |
| 4. | It is important for me to search bibliographic databases to be an effective clinician. | | | | 7 | | 6 | | 5 | | 4 | | 3 | | 2 | 1 |
| 5. | It is important for me to critically appraise research papers to be an effective clinician. | | | | 7 | | 6 | | 5 | | 4 | | 3 | | 2 | 1 |
| 6. | Evidence and patients are equally important to making clinical decisions. | | | | 7 | | 6 | | 5 | | 4 | | 3 | | 2 | 1 |
|  |  | |  |  | |  | |  | |  | |  | |  | | |
| 7. | What % of your patient decisions are based on evidence from clinical research?  ___________% | | | | | | | | | | | | | | | |

8. How frequently do you have questions about managing your patients that might require research evidence to answer?

(i) _______ times per day (on average)

(ii) _______ times per week (on average)

|  | | | | | | | | | | | | **Every day** | | | | **Every other day** | | | | **Every week** | | | | **Every month** | | | | **Never** | | **Other**  **(please specify)** | | |
| --- | --- | --- | --- | --- | --- | --- | --- | --- | --- | --- | --- | --- | --- | --- | --- | --- | --- | --- | --- | --- | --- | --- | --- | --- | --- | --- | --- | --- | --- | --- | --- | --- |
| 9. | How frequently do you access clinical research evidence *in general*? | | | | | | | | | | | 5 | | | | 4 | | | | 3 | | | | 2 | | | | 1 | | __________ | | |
| 10. | How frequently do you access clinical research evidence *from a textbook*? | | | | | | | | | | | 5 | | | | 4 | | | | 3 | | | | 2 | | | | 1 | | __________ | | |
| 11. | How frequently do you access clinical research evidence *from original research papers*? | | | | | | | | | | | 5 | | | | 4 | | | | 3 | | | | 2 | | | | 1 | | __________ | | |
| 12. | How frequently do you access clinical research evidence *from the Cochrane database*? | | | | | | | | | | | 5 | | | | 4 | | | | 3 | | | | 2 | | | | 1 | | __________ | | |
| 13. | How frequently do you access clinical research evidence *from secondary sources such as ACP Journal Club, the journal Evidence-Based Medicine, POEMs (Patient-oriented evidence that matters) or CATs (Critically appraised topics)*? | | | | | | | | | | | 5 | | | | 4 | | | | 3 | | | | 2 | | | | 1 | | __________ | | |
|  |  | | | | | | | | | | |  | | | | |  | | | | |  | | |  | | | |  |  | | |
| 14. | On average, how often do you now look up evidence immediately before, or during patient treatment visit?  _______ hours/week | | | | | | | | | | | | | | | | | | | | | | | | | | | | | | | |
| 15. | How many hours/week do you spend looking up evidence?  __________hours/week | | | | | | | | | | | | | | | | | | | | | | | | | | | | | | | |
| 16. | How many hours/week do you spend reading new research evidence?  __________hours/week | | | | | | | | | | | | | | | | | | | | | | | | | | | | | | | |
| 17. | How much has the use of evidence-based practice affected your clinical decisions?   | Completely | A lot | Moderately | Somewhat | A little | Not at all | | --- | --- | --- | --- | --- | --- | | | | | | | | | | | | | | | | | | | | | | | | | | | | | | | | |
|  | 6 | | 5 | | | 4 | | | 3 | | | | 2 | | | | | | 1 | | | | | | |  | | | | | | |
| 18. | How much has the use of evidence-based practice affected your patient outcomes?   | Completely | A lot | Moderately | Somewhat | A little | Not at all | | --- | --- | --- | --- | --- | --- | |  |  |  |  |  |  | | | | | | | | | | | | | | | | | | | | | | | | | | | | | | | |  |
|  |  | | | | | | | | | | | | | | | | | | | | | | | | | | | | | | |  |
|  | 6 | | | 5 | | | 4 | | | 3 | | | | 2 | | | | | | | 1 | | | | | |  | | | | | |
| 19. | How often does new research evidence result in a change in your practice? | | | | | | | | | | | | | | | | | | | | | | | | | | | | | | |  |
|  | All the time | | Regularly | | | Frequently | | | Occasionally | | | | Almost Never | | | | | | Never | | | | | | |  | | | | | |  |
|  | 6 | | 5 | | | 4 | | | 3 | | | | 2 | | | | | | 1 | | | | | | |  | | | | | |  |
|  |  | | | | | | | | | | | | | | | | | | | | | | | | | | | | | | |  |
|  |  |  | | | |  | | | |  | | | | |  | | | | | | | |  | | |  | | | | | |  |
|  |  |  | | | |  | | | |  | | | | |  | | | | | | | |  | | |  | | | | | |  |
| 20. | How much confidence do you have in your clinical decision-making? | | | | | | | | | | | | | | | | | | | | | | | | | | | | | | |  |
|  | A lot | | | | A moderate  amount | | | Some | | | A little | | | | | | | None at all | | | | | | | |  | | | | | |  |
|  | 5 | | | | 4 | | | 3 | | | 2 | | | | | | | 1 | | | | | | | |  | | | | | |  |
|  |  |  | | | |  | | | |  | | | | |  | | | | | | | |  | | |  | | | | |  | |

| The following questions are asking about your personal opinion about EBP. There are no correct answers.  Please indicate how much you agree/disagree with the following statements. | | | | | | | | |
| --- | --- | --- | --- | --- | --- | --- | --- | --- |
|  |  | **Strongly Agree** | **Moderately Agree** | **Somewhat Agree** | **Neutral** | **Somewhat Disagree** | **Moderately Disagree** | **Strongly Disagree** |
| 21. | Evidence-based practice is “cook-book” rehabilitation that disregards clinical experience. | 7 | 6 | 5 | 4 | 3 | 2 | 1 |
| 22. | It is easy to find the research. | 7 | 6 | 5 | 4 | 3 | 2 | 1 |
| 23. | Evidence-based practice takes too much time. | 7 | 6 | 5 | 4 | 3 | 2 | 1 |
| 24. | Evidence-based practice ignores the “art” of clinical practice | 7 | 6 | 5 | 4 | 3 | 2 | 1 |
| 25. | Previous clinical experience is more important than research findings in choosing the best treatment available for a patient. | 7 | 6 | 5 | 4 | 3 | 2 | 1 |
| 26. | Evidence-based practice should be an integral part of clinical practice. | 7 | 6 | 5 | 4 | 3 | 2 | 1 |
| 27. | From my personal observation and experience, evidence-based practice is being used currently by my colleagues. | 7 | 6 | 5 | 4 | 3 | 2 | 1 |
| 28. | I use evidence-based practice because it improves patient outcomes. | 7 | 6 | 5 | 4 | 3 | 2 | 1 |
| 29. | I use evidence-based practice because I believe in it. | 7 | 6 | 5 | 4 | 3 | 2 | 1 |
| 30. | I use evidence-based practice because my colleagues do. | 7 | 6 | 5 | 4 | 3 | 2 | 1 |
| 31. | I don’t use evidence-based practice because I don’t have time. | 7 | 6 | 5 | 4 | 3 | 2 | 1 |
| 32. | I don’t use evidence-based practice because it is difficult to change. | 7 | 6 | 5 | 4 | 3 | 2 | 1 |
| 33. | I don’t use evidence-based practice for another reason (specify): ____________________________________________________ | 7 | 6 | 5 | 4 | 3 | 2 | 1 |

| **Thank you for taking the time to complete this survey.** |
| --- |
