# Supplementary material for: A modified evidence-based practice- knowledge, attitudes, behaviour and decisions/outcomes questionnaire is valid across multiple professions involved in pain management
Source: BMC Med Educ. 2014 Dec 14;14:263. doi: 10.1186/s12909-014-0263-4 (PMC4272818; doi:10.1186/s12909-014-0263-4)
Supplement: Additional file 2: — Comparison of EBP-KABO to KAB questionnaire developed by Johnson et al. [file 12909_2014_263_MOESM2_ESM.docx]

Appendix 2. Comparison of EBP-KABO to KAB questionnaire developed by Johnson et al.

| **Original KAB Items** | **KAB-EBP Question –rewritten based on pilot study and analyzed in this study** |
| --- | --- |
| **Domain of Knowledge** | **Domain of Knowledge** |
| --- | 1. I am confident in my ability to use evidence-based practice (**new items**) |
| Practicing evidence-based medicine increases the certainty that the proposed treatment is effective | 2. Using evidence-based practice increases the certainty that the proposed treatment is effective |
| Research using clinical trials is generally more reliable than research using the observational method | 3. Research using clinical trials is generally more reliable than research using the observational method **(same as KAB)** |
| Effective searching skills ⁄ easy access to bibliographic databases and evidence sources are essential to practicing evidence-based medicine | 4.It is important for me to search bibliographic databases to be an effective clinician |
| Evidence-based medicine requires the use of critical appraisal skills to ensure the quality of all the research papers retrieved | 5. It is important for me to critically appraise research papers to be an effective clinician. |
| Critically appraised evidence should be appropriately applied to the  patient using clinical judgment and experience | 6. Evidence and patients are equally important to making clinical decisions. |
| In your opinion, how much of clinical practice currently, on average, is grounded in scientific evidence that looks specifically at patient outcomes (i.e. morbidity and mortality)? | 7. What % of your patient decisions are based on evidence from clinical research? |
| **Domain of personal application and use** | **Domain of (EBP) behavior** |
| How frequently do you access medical evidence in general? | 9. How frequently do you access clinical research evidence *in general*? **(same as KAB)** |
| How frequently do you access rehabilitation evidence from a textbook? | 10. How frequently do you access clinical research evidence *from a textbook*? **(same as KAB)** |
| How frequently do you access medical evidence from original research papers? | 11. How frequently do you access clinical research evidence *from original research papers*? **(same as KAB)** |
| How frequently do you access medical evidence from the Cochrane database? | 12. How frequently do you access clinical research evidence *from the Cochrane database*? **(same as KAB)** |
| How frequently do you access medical evidence from secondary sources such as the ACP Journal Club, the Journal of Evidence-Based Medicine, POEMs (Patient Oriented Evidence that Matters) or CATs (Critically Appraised Topics)? | 13. How frequently do you access clinical research evidence *from secondary sources such as ACP Journal Club, the journal Evidence-Based Medicine, POEMs (Patient-oriented evidence that matters) or CATs (Critically appraised topics)*? |
| Compared to one year ago, on average, how often do you now look up evidence (i.e. information or content materials relating to the patient's condition) during or after clerking each patient on the ward or in the clinic  (i) _______ times per patient admission (inpatient)  (ii) _______ times per patient visit (outpatient) | 14. On average, how often do you now look up evidence immediately before, or during patient treatment visit?  _______ hours/week |
| Compared to one year ago, on average, how much time do you now spend every day finding or looking up evidence (or content material)? [This does NOT include your study time -- ONLY the time it takes you to retrieve the material]  __________mins | 15. How many hours/week do you spend looking up evidence?  __________hours/week |
| See above | 16. How many hours/week do you spend reading new research evidence?  __________hours/week |
| **Domain of future use** | **Domain of (EBP) decisions and outcomes** |
| In your opinion and judgment, how much has the practice of evidence-based practice, on average, affected the management or outcome of the patients you have treated? | 17. How much has the use of evidence-based practice affected your clinical decisions? |
| In your opinion and judgment, how much has the use of evidence-based practice, on average, affected the management or outcome of the patients you have treated? | 18. How much has the use of evidence-based practice affected your patient outcomes? |
| How much has the use of evidence-based practice changed the way you learn | 19. How often does new research evidence result in a change in your practice? |
| **Domain of attitude toward EBP** | **Domain of attitudes (towards EBP)** |
| How much confidence do you have in your clinical decision-making | 20. How much confidence do you have in your clinical decision-making |
| Evidence-based medicine is cook-book medicine that disregards clinical experience | 21. Evidence-based practice is “cook-book” rehabilitation that disregards clinical experience. |
| It is easy to find the evidence in order to practice evidence-based medicine | 22. It is easy to find the research. |
| Evidence-based medicine takes too much time for busy medical students. | 23. Evidence-based practice takes too much time. |
| Evidence-based medicine ignores the art of medicine | 24. Evidence-based practice ignores the “art” of clinical practice. **(same as KAB)** |
| Previous work experience is more important than research findings in  choosing the best treatment available for a patient | 25. Previous clinical experience is more important than research findings in choosing the best treatment available for a patient. **(same as KAB)** |
| Evidence-based practice should be an integral part of the undergraduate medical curriculum | 26. Evidence-based practice should be an integral part of clinical practice. |
| From your personal observation and experience, evidence-based practice is being used currently in Hong Kong. | 27. From my personal observation and experience, evidence-based practice is being used currently by my colleagues. |
| --- | 28. I use evidence-based practice because it improves patient outcomes. **(new item)** |
| I don’t use evidence-based practice because I don’t believe in it. | 29. I use evidence-based practice because I believe in it. |
| I don’t use evidence-based practice because my colleagues don’t | 30. I use evidence-based practice because my colleagues do. |
| I don’t use evidence-based practice because I don’t have time. | 31. I don’t use evidence-based practice because I don’t have time. |
| I don’t use evidence-based practice because of personal procrastination in changing old habits. | 32. I don’t use evidence-based practice because it is difficult to change. |
| Other reason(s): (please specify) | 33. I don’t use evidence-based practice for another reason (specify): |
